# Association between Cholecystectomy and the Incidence of Pancreaticobiliary Cancer after Endoscopic Choledocholithiasis Management

**DOI:** 10.3390/cancers16050977

**Published:** 2024-02-28

**Authors:** Chi-Chih Wang, Jing-Yang Huang, Li-Han Weng, Yao-Chun Hsu, Wen-Wei Sung, Chao-Yen Huang, Chun-Che Lin, James Cheng-Chung Wei, Ming-Chang Tsai

**Affiliations:** 1 Division of Gastroenterology and Hepatology, Department of Internal Medicine, Chung Shan Medical University Hospital, Taichung City 40201, Taiwan; bananaudwang@gmail.com (C.-C.W.); b101099039@gmail.com (L.-H.W.); forest65@csmu.edu.tw (C.-C.L.); 2 Institute of Medicine, Chung Shan Medical University, Taichung 40201, Taiwan; wchinyang@gmail.com (J.-Y.H.); flutewayne@gmail.com (W.-W.S.); 3 School of Medicine, Chung Shan Medical University, Taichung 40201, Taiwan; moongenius0913@gmail.com; 4 Center for Health Data Science, Chung Shan Medical University, Taichung 40201, Taiwan; 5 Center for Liver Diseases and Center for Clinical Trials, E-Da Hospital, Kaohsiung, Taiwan; holdenhsu@gmail.com; 6 School of Medicine, I-Shou University, Kaohsiung 84001, Taiwan; 7 Department of Emergency Medicine, Chung Shan Medical University Hospital, Taichung 40201, Taiwan; 8 Department of Allergy, Immunology, and Rheumatology, Chung Shan Medical University Hospital, Taichung 40201, Taiwan

**Keywords:** endoscopic retrograde cholangiopancreatography, cholecystectomy, pancreaticobiliary cancer, ampullary cancer, future cancer risk

## Abstract

**Simple Summary:**

The majority of the current evidence shows that people who have been accepted to receive cholecystectomy have higher hepatobiliary and pancreatic cancer risk. Meanwhile, strong evidence showed that cholecystectomy can reduce recurrent biliary events after endoscopic treatment for choledocholithiasis, and we suggest on-site or interval cholecystectomy in such patients. We need to explore the true risk of the pancreaticobiliary system after the endoscopic management of choledocholithiasis.

**Abstract:**

(1) Background: Previous studies have raised concerns about a potential increase in pancreaticobiliary cancer risk after cholecystectomy, but few studies have focused on patients who undergo cholecystectomy after receiving endoscopic retrograde cholangiopancreatography (ERCP) for choledocholithiasis. This study aims to clarify cancer risks in these patients, who usually require cholecystectomy, to reduce recurrent biliary events. (2) Methods: We conducted a nationwide cohort study linked to the National Health Insurance Research Database, the Cancer Registry Database, and the Death Registry Records to evaluate the risk of pancreaticobiliary cancers. All patients who underwent first-time therapeutic ERCP for choledocholithiasis from 2011 to 2017 in Taiwan were included. We collected the data of 13,413 patients who received cholecystectomy after endoscopic retrograde cholangiopancreatography and used propensity score matching to obtain the data of 13,330 patients in both the cholecystectomy and non-cholecystectomy groups with similar age, gender, and known pancreaticobiliary cancer risk factors. Pancreaticobiliary cancer incidences were further compared. (3) Results: In the cholecystectomy group, 60 patients had cholangiocarcinoma, 61 patients had pancreatic cancer, and 15 patients had ampullary cancer. In the non-cholecystectomy group, 168 cases had cholangiocarcinoma, 101 patients had pancreatic cancer, and 49 patients had ampullary cancer. The incidence rates of cholangiocarcinoma, pancreatic cancer, and ampullary cancer were 1.19, 1.21, and 0.3 per 1000 person-years in the cholecystectomy group, all significantly lower than 3.52 (*p* < 0.0001), 2.11 (*p* = 0.0007), and 1.02 (*p* < 0.0001) per 1000 person-years, respectively, in the non-cholecystectomy group. (4) Conclusions: In patients receiving ERCP for choledocholithiasis, cholecystectomy is associated with a significantly lower risk of developing pancreaticobiliary cancer

## 1. Introduction

Cholecystectomy (CCY) is an important treatment [1] of choledocholithiasis after endoscopic treatment in different types of biliary events, including cholecystitis, cholangitis, and gallstone pancreatitis. This problem has become important due to obesity and is an important risk factor for cholelithiasis, which increases in prevalence in young-age people [2]. There is extensive evidence that CCY, after endoscopic retrograde cholangiopancreatography (ERCP) management for choledocholithiasis, can decrease future recurrent biliary events [3,4,5,6]. However, previous studies have shown that CCY might increase cancer risks in total liver cancers [7,8] hepatocellular carcinoma (HCC) [9,10], intra-hepatic cholangiocarcinoma (ICC) [9], extra-hepatic cholangiocarcinoma (ECC) [11], ampullary cancer (AVC) [11,12], and pancreatic duct adenocarcinoma (PDC) [7,11,12,13]. Because evidence of biliary tract malignancy could be caused by the condition of chronic inflammation resulting from cholestasis [14,15], CCY should be associated with a decreased incidence of bile duct cancers after patients undergo therapeutic ERCP for choledocholithiasis. However, conflicting results suggest a lack of direct association between CCY and hepatobiliary system cancers [16,17]. Meanwhile, laparoscopic cholecystectomy and common bile duct exploration (LCBDE) was shown to resolve this problem in one step without destroying the structure of Ampulla of Vater in the management of choledocholithiasis compared with CCY after ERCP [18]. The current evidence for the correlation between increased cancer risks in the pancreatic and hepatobiliary system and CCY or endoscopic sphincterotomy (ES) [19] is not completely clear. The problem in establishing a cause-and-effect relationship between PDAC, hepatobiliary system malignancy, and CCY is the procedure per se or the chronic inflammation environment caused by gallstone disease (i.e., the reason to perform CCY).

Biliary tract cancers, which can be divided into ICC, ECC, and AVC, are lethal diseases in the biliary system [20]. Our previous population-database cohort study showed that CCY was associated with a lower risk of cholangiocarcinoma in patients who underwent ERCP for bile duct stones management [21], but the evidence was not strong owing to the limited number of further cholangiocarcinoma cases. Therefore, we conducted a large-scale whole population-database cohort study in Taiwan and provided detailed confounding factor adjustments to clarify the association between CCY and the risk of pancreaticobiliary cancers after the ERCP management of choledocholithiasis and acute cholangitis.

## 2. Materials and Methods

We conducted a nationwide cohort study including whole population data, which comprised about 23 million beneficiaries who were enrolled in the Health Insurance Program in Taiwan from 1 January 2011 to 31 December 2018. The data were accessed from Taiwan’s Health and Welfare Data Science Center (HWDSC). This center maintains many nationwide databases in Taiwan for the purpose of academic research [22]. We linked three nationwide databases, the National Health Insurance Research Database (NHIRD), the Cancer Registry Database, and the Death Registry Records, to evaluate the risk of pancreaticobiliary cancers, including ICC, ECC, AVC, and PDAC in choledocholithiasis patients after ES or endoscopic papillary balloon dilatation (EPBD). The information in the registry data of inpatient and outpatient visits was identified, including personal demographics, diagnosis of potential disease, prescriptions, and surgeries from the NHIRD. The Cancer Registry Database was established in 1979 (https://twcr.tw/), and it contains a high-quality database of the validated registry framework. We extracted data on the date of cancer diagnosis and the cancer site from the Cancer Registry Database. The Death Registry Records were used to check the survival status, date of death, and cause of death. All study individuals were provided with a hashed and unique personal identification number to link the data between these nationwide databases. The primary outcome in this study is further biliary cancer risk, while the secondary outcome is PDAC risk after CCY in patients, whoever accepted ERCP choledocholithiasis management.

The Human Research Ethics Committee of the Institutional Review Board of Chung Shan Medical University Hospital approved our study (CS1-22080) on 27 June 2022. This study was sponsored by the Chung Shan Medical University Hospital Research Program (CSH-2021-C-006, CSH-2022-C-044).

### 2.1. Definition of Study Population

We identified patients hospitalized for the diagnosis of choledocholithiasis (ICD-9 codes: 574.3, 574.4, 574.5, 574.6, 574.7, 574.8, and 574.9), cholangitis (ICD-9 codes: 576.1 and 576.2), and treated with therapeutic ERCP (ES [procedure code: 56031B and 56033B] or EPBD [procedure code: 56032B]) for the first time, who were also identified by procedure codes of lithotripsy (28008B, 28035B) between 2011 and 2017 and followed the outcomes of these patients until 31 December 2018. Initially, 55,459 patients were selected, and the patients who had missing demographic data, were aged <18 years at admission, and had received CCY before admission were excluded. After the first exclusion, there were 14,068 patients who had received CCY within 2 months [23] after the first admission as the CCY group and 38,259 patients who did not have CCY throughout the study period as the comparison group. We defined the index date as the date 2 months after initial admission to avoid potential surveillance or detection bias. Furthermore, we excluded patients who died or had biliary tract malignancy before the index date. The 13,413 and 33,068 eligible cases were divided into CCY and non-CCY cohorts, respectively.

**Figure 1:**
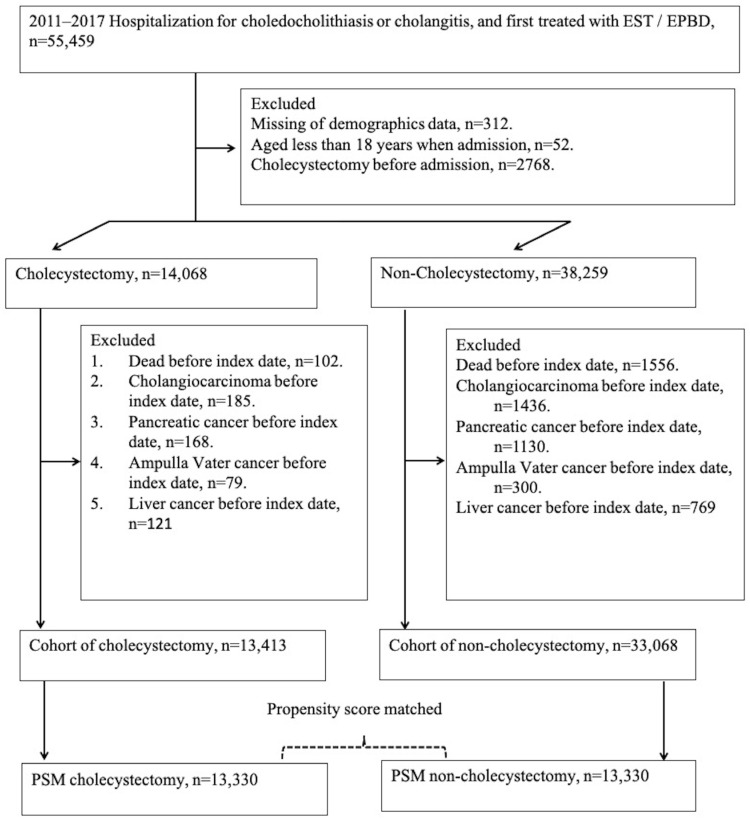
Flowchart of patient selection.

An additional analysis of propensity score matching (PSM) was performed to reduce the confounding bias after balancing the measured characteristics between the study groups [24]. We also provide the original baseline characteristics among study groups in Supplementary Table S1. The propensity score was estimated as the probability of the treatment of CCY by using logistic regression, and the covariates included were index year, baseline demographics (including sex, age, urbanization, and insured category), and pancreaticobiliary cancer risk factors (including chronic hepatitis B infections [CHB] [25], chronic hepatitis C [10] [CHC], Helicobacter pylori [HP] infection [26,27], diabetes mellitus [28,29] [DM], chronic kidney disease [CKD], choledochal cyst disease [30,31], inflammatory bowel diseases [IBD], and liver cirrhosis [LC] [8,32]). There were some rare diagnoses, which may increase biliary cancer risks, that were never recorded in our analysis, such as primary sclerosing cholangitis [33] and specific parasite infection [34]. We used the PSMATCH procedure in the SAS software version 9.4, the algorithm of greedy nearest neighbor matching, and non-replacement paired within 0.01 caliper widths [35]. Finally, 13,330 pairs of PSM CCY and non-CCY patients were selected for analysis (Figure 1).

### 2.2. Definition of Study Covariates

Baseline demographics, including age, sex, urbanization, and insured category, were identified based on the information at the index date. Age was calculated in years between the birth and index dates, and we classified patients into the age groups of 18 to <50, 50 to <60, 60 to <70, and ≥70 years. The risk factors of pancreaticobiliary cancers, including CHB, CHC, HP infection, DM, CKD, congenital cystic disease of the liver, IBD, and LC, were identified based on the ICD-9-CM codes listed in Supplementary Table S2. The baseline characteristics among study groups after PSM are shown in Table 1.

### 2.3. Identification of Study Events

The subsequent pancreaticobiliary cancer was identified from the information in the Cancer Registry Database. The patients who were newly diagnosed with cholangiocarcinoma (ICC and ECC), PDAC, and AVC were ascertained using ICD-9 codes (Supplementary Table S2), while lung cancer was selected for potential bias detection.

### 2.4. Statistical Analysis

We used the absolute standardized difference (ASD) [36] to compare the baseline covariates between the groups in this large-sample observational study. The characteristics were balanced when the ASD was <0.1. We conducted survival models to evaluate the association between CCY and the risk of pancreaticobiliary cancers. The incidence rate and 95% confidence interval (CI) were calculated by considering the Poisson distribution. All study individuals were followed from the index date until the occurrence of the study event. The censoring point included the death of patients or the end of the study (31 December 2018). Kaplan–Meier survival curves were plotted to compare the 7-year cumulative probability of developing cholangiocarcinoma (including ICC and ECC), PDAC, AVC, or lung cancer among the CCY and non-CCY cohorts, with a median follow-up period of 3.9 years. A log-rank test was performed to determine the overall homogeneity of the hazard rate functions among the study groups. After the proportional hazard assumption was tested, univariate and multivariable Cox proportional hazards regressions were used to estimate the hazard ratio (HR) of the exposure to CCY on the risk of cholangiocarcinoma, PDAC, AVC, and lung cancer (Table 2). We considered the covariates, including index year, baseline demographics, and known pancreaticobiliary cancer risks, which were described in the definition of the study population in the multivariable regression. The competing HR was estimated using the sub-distribution Fine–Gray regression approach, wherein mortality was considered a competing event. A subgroup analysis and an interaction effect test were performed to evaluate the effects of sex and age in different stratifications using multivariate Cox regression. When considering the possible incubation period from exposure to the diagnosis of cancer, we performed the sensitivity analysis using the landmark time at 24 months after the index date. We excluded individuals who were followed for less than 24 months in the landmark analysis. All statistical analyses were conducted using SAS version 9.4 (SAS Institute, Cary, NC, USA). A significance level of 0.05 was used for the hypothesis test.

## 3. Results

We collected 13,330 cases who received CCY after ES or EPBD for choledocholithiasis or cholangitis management. We provided another 13,330 cases without CCY after PSM under similar clinical conditions as the control group (Figure 1). In the baseline covariates, the ASD in the index year, gender, age, urbanization, and insured category, which were all less than 0.1, means balanced baseline characters between the CCY and non-CCY groups. Meanwhile, there were no statistically significant differences in the known risk factors for pancreaticobiliary cancer, such as CHB, CHC, Helicobacter infection, diabetes mellitus, CKD, congenital cystic disease of the liver, IBD, and LC, between the CCY and non-CCY cohorts. Detailed information is shown in Table 1.

The results showed that 168 patients had cholangiocarcinoma in the non-CCY group, while 60 cases had cholangiocarcinoma in the CCY group. There were 3.52, 2.45, and 1.09 cases per 1000 person-years of cholangiocarcinoma, ICC, and ECC in the non-CCY group and 1.19, 0.91, and 0.48 cases per 1000 person-years of cholangiocarcinoma, ICC, and ECC, respectively, in the CCY group. The competing HR in the multivariable regression, considering the covariates, including index year, baseline demographics (including sex, age, urbanization, and insured category), and risk factors for pancreaticobiliary cancers (including CHB, CHC, HP infection, DM, CKD, congenital cystic disease of the liver, IBD, and LC), revealed an adjusted HR of the CCY group of 0.34 (0.25–0.46), 0.37 (0.26–0.52), 0.44 (0.27–0.72) in cholangiocarcinoma, ICC, and ECC, respectively, compared with the risks of the non-CCY group.

There were 101 cases of PDAC in the follow-up period, which resulted in an incidence rate of 2.11 (1.74–2.57) per 1000 person-years in the non-CCY group, while there were 61 cases of PDAC with an incidence rate of 1.21 (0.94–1.56) per 1000 person-years in the CCY group. The adjusted HR of PDAC in the CCY group compared with the non-CCY group was 0.58 (0.42–0.79), with a *p* value of 0.0007. Similar results were noticed in the AVC, which showed that the adjusted HR of the AVC in the CCY group compared with the non-CCY group was 0.30 (0.17–0.53), with a *p* value of <0.0001. For potential bias detection, we chose lung cancer, which is supposed to have no relationship with CCY, to calculate the adjusted HR between the CCY and non-CCY cohorts. The adjusted HR of lung cancer was 0.86 (0.61–1.21) in the CCY group compared with the non-CCY group, which showed a non-significant difference, with a *p* value of 0.3737. Detailed information is provided in Table 2. We provided the pancreaticobiliary cancer risk in the study groups before PSM in Supplementary Table S3.

**Figure 2:**
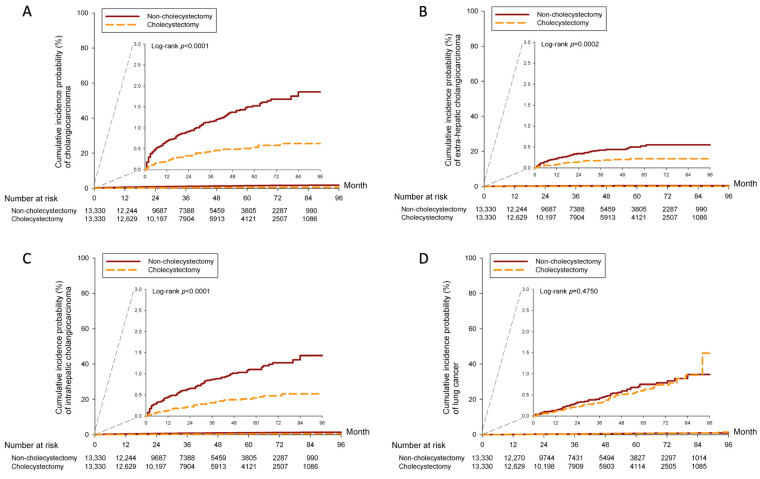
KM curves of incidence probability of (**A**) cholangiocarcinoma, (**B**) extra-hepatic cholangiocarcinoma, (**C**) intrahepatic cholangiocarcinoma, and (**D**) lung cancer.

**Figure 3:**
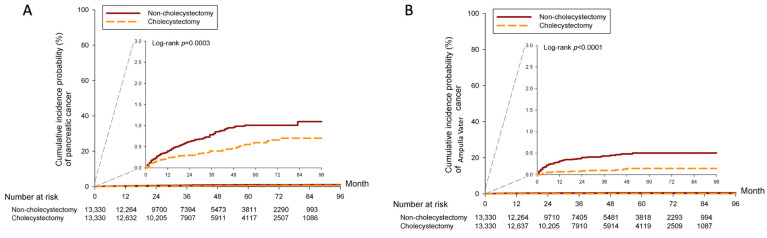
KM curves of incidence probability of (**A**) pancreatic cancer and (**B**) ampullary cancer.

Kaplan–Meier curves were plotted to compare the 7-year cumulative probability of developing cholangiocarcinoma, ICC, ECC, and lung cancer in Figure 2. The cumulative incidence probability of cholangiocarcinoma, ECC, and ICC was significantly higher in the non-CCY group than in the CCY group, with *p* values of *p* < 0.0001, *p* = 0.0002, and *p* < 0.0001, respectively. The Kaplan–Meier curve of lung cancer incidence probability was similar (*p* = 0.4750) between the non-CCY and CCY groups in Panel D, Figure 2. The cumulative incidence probability of PDAC, which is demonstrated in Panel A, Figure 3, is significantly higher in the non-CCY group (*p* = 0.0003), while the AVC risk is also higher (*p* < 0.0001) in the non-CCY group (Panel B, Figure 3).

We performed the sensitivity analysis using the landmark time at 24 months after the index date by excluding individuals who were followed for less than 24 months in the landmark analysis. The competing adjusted hazard ratios of the CCY group compared with that of the non-CCY group were 0.30 (0.17–0.51) in cholangiocarcinoma, 0.44 (0.25–0.75) in ICC, 0.46 (0.18–1.14) in ECC, 0.84 (0.47–1.48) in PDAC, 0.48 (0.16–1.40) in AVC, and 1.05 (0.65–1.68) in lung cancer when individuals who were followed for less than 24 months were excluded. Detailed information is presented in Table 3. The original results before PSM showed similar conditions in Supplementary Table S4.

## 4. Discussion

This is the first nationwide whole-population study to evaluate the cancer risk of CCY in patients after choledocholithiasis or cholangitis managed with therapeutic ERCP. The inclusion criteria used both the diagnosis code and procedure code of ES or EPBD combined with lithotripsy to ensure disease accuracy and balance the heterogeneous condition of choledocholithiasis in both CCY and non-CCY groups in order to minimize the limitations inherent to retrospective research. These patients usually need CCY to reduce recurrent biliary events in the rest of their lifetimes, but the decision becomes more difficult because of prior studies reporting that CCY could possibly increase cancer risk. In our study, we found that the competing adjusted hazard ratios in the CCY group were 0.34 (95% CI, 0.25–0.46), 0.37 (95% CI, 0.26–0.52), 0.44 (95% CI, 0.27–0.72), 0.58 (95% CI, 0.42–0.79), and 0.30 (95% CI, 0.17–0.53) for cholangiocarcinoma, ICC, ECC, PDAC, and AVC, respectively, compared with the risk in the non-CCY group. Instead of increasing cancer risk, CCY was associated with a significantly lower risk of pancreaticobiliary cancer by 42–70% (ICC 63% reduction, ECC 56%, PDAC 42%, and AVC 70%). These results remained consistent in the sensitivity tests, including the landmark analysis. Across multiple analytical methods, no evidence was found to suggest that CCY was associated with elevated risks of ECC, PDAC, or AVC.

There is very limited clinical evidence focusing on cancer risks after CCY in patients with choledocholithiasis, which was previously managed by therapeutic ERCP. In pancreatic and hepatobiliary systems, previous cohort studies demonstrated that patients who received CCY had higher cancer risks of overall liver cancers [7], HCC [9,10], ICC [9], ECC [11], AVC [11,12], and PDAC [7,11,12]. Meta-analysis studies also supported the increased risk of liver cancer [37,38] and PDAC [13] in patients with gallstone disease after CCY. Only one review showed no association between CCY and cancer risk in the hepatobiliary system after a strict quality checkup [17]. In contrast, our study exhibits opposite results: the incidence of cholangiocarcinoma, ICC, ECC, AVC, and PDAC were all significantly lower and reduced in crude, adjusted, and competing adjusted hazard ratios after CCY subsequent to therapeutic ERCP choledocholithiasis treatment. There might be some major issues in previous literature, such as earlier studies using historical comparisons [11,12] and case-control study designs [7]. Although other studies [9,39] have performed the adjustment of gallstone disease, the disease severity of cholelithiasis in patients who received CCY and in patients who need no intervention is still different from the background inflammation situation.

While we focused on cholangiocarcinoma risk, a previous large-scale Swedish database study [40] showed increased ICC and ECC risks in the CCY group compared with the non-CCY group, while the risk of these tumors is reduced back to the level of the background population more than 10 years after CCY. However, this study used patients with non-symptomatic gallstone disease as a control group, which may be confounded by the disease severity of gallstone disease. A recent systemic review [41] showed that CCY was associated with a significant 54% increase in the risk of cholangiocarcinoma, especially in the ECC, in comparison with healthy controls. The updated study, which focused on complicated gallstone disease patients, showed that CCY slightly decreased cholangiocarcinoma risk [42], but this study still used age-matched and sex-matched controls without further detailed confounding factors adjustments. The problems of heterogenous disease severity and inflammatory conditions due to cholelithiasis in the CCY group and in the pure gallstone disease group/normal population existed in almost all previous articles. Our study design directly discussed the future cancer risk in patients who received therapeutic ERCP intervention for choledocholithiasis in both patients who received CCY or chose not to receive CCY. We believe this study design can help clarify the truest risk of CCY for pancreaticobiliary cancer incidence in patients who have undergone ES or EPBD for choledocholithiasis and need CCY to reduce recurrent biliary events in the rest of their lifetime.

### Limitations

There are some limitations to our study. First, our study, like most retrospective database studies, had some confounding factors, such as environmental risk exposure, personal habits, and exact family history, which cannot be corrected and may lead to some clinical bias. Second, although the longest follow-up period is 7.6 years, the mean follow-up time in our cohort study is 3.9 years, which is relatively short. However, we cannot expand the observational period due to all the data being kept in HWDSC in accordance with our regulations in Taiwan. Third, the general characteristics of the CCY and non-CCY groups were less homogenous in the original data, which required PSM to balance the background parameters. However, the PSM process itself led to a loss of study samples and may have reduced statistical power, especially in the sensitivity analysis, with the exclusion of patients followed up for less than 2 years. Fourth, we didn’t evaluate further the cancer risk in patients who accepted LCBDE because we seldom performed this procedure routinely in Taiwan. As a result, our results should be applied with caution to the patients who underwent LCBDE.

## 5. Conclusions

In patients who underwent therapeutic ERCP for choledocholithiasis or cholangitis, CCY was not associated with an excessive risk of pancreaticobiliary cancer. Instead, the relationship between CCY and the subsequent development of pancreaticobiliary cancer post-ERCP appeared to be protective.

## Figures and Tables

**Table 1:** Baseline characteristics among study groups after propensity score matching (PSM).

	Non-Cholecystectomy	Cholecystectomy	ASD
*n*	13,330	13,330	
Index year			0.000
2011, 2012	2932 (22.00%)	2911 (21.84%)	
2013, 2014	3615 (27.12%)	3585 (26.89%)	
2015, 2016	4144 (31.09%)	4189 (31.43%)	
2017, 2018	2639 (19.80%)	2645 (19.84%)	
Sex			0.004
Male	7111 (53.35%)	7047 (52.87%)	
Female	6219 (46.65%)	6283 (47.13%)	
Age			0.000
<50	3662 (27.47%)	3636 (27.28%)	
50–60	2516 (18.87%)	2510 (18.83%)	
60–70	2934 (22.01%)	2988 (22.42%)	
≥70	4218 (31.64%)	4196 (31.48%)	
Urbanization			0.000
High urbanization	3928 (29.47%)	3872 (29.05%)	
Moderate urbanization	4147 (31.11%)	4160 (31.21%)	
Developing town	2128 (15.96%)	2094 (15.71%)	
General town	1833 (13.75%)	1848 (13.86%)	
Aged town	368 (2.76%)	367 (2.75%)	
Agriculture town	605 (4.54%)	636 (4.77%)	
Village	321 (2.41%)	353 (2.65%)	
Insured category			0.083
Government	701 (5.26%)	721 (5.41%)	
Privately held company	7235 (54.28%)	7124 (53.44%)	
Agricultural organizations	2379 (17.85%)	2387 (17.91%)	
Low-income	90 (0.68%)	103 (0.77%)	
Non-labor force	2737 (20.53%)	2776 (20.83%)	
Others	188 (1.41%)	219 (1.64%)	
Co-morbidity			
Chronic hepatitis B	1029 (7.72%)	1134 (8.51%)	0.029
Chronic hepatitis C	406 (3.05%)	465 (3.49%)	0.025
Helicobacter infection	397 (2.98%)	466 (3.50%)	0.029
Diabetes mellitus	4222 (31.67%)	4210 (31.58%)	0.002
CKD	1276 (9.57%)	1270 (9.53%)	0.002
Congenital cystic disease of liver	93 (0.70%)	90 (0.68%)	0.003
Inflammatory bowel diseases	375 (2.81%)	427 (3.20%)	0.023
Liver cirrhosis	516 (3.87%)	510 (3.83%)	0.002

ASD, absolute standardized difference. CKD, chronic kidney disease.

**Table 2:** Risks of cancer incidence among patients with EST/EPBD after PSM.

	Non-Cholecystectomy	Cholecystectomy	*p* Value
Cholangiocarcinoma			
Observed person-years	47,718.8	50,262.1	
Incident cases	168	60	
Incidence rate †	3.52 (3.03–4.1)	1.19 (0.93–1.54)	
Crude HR (95% CI)	Reference	0.34 (0.26–0.46)	<0.0001
Adjusted HR (95% CI)	Reference	0.34 (0.25–0.46)	<0.0001
ICC			
Observed person-years	47,768.3	50,276.6	
Incident cases	117	46	
Incidence rate †	2.45 (2.04–2.94)	0.91 (0.69–1.22)	
Crude HR (95% CI)	Reference	0.38 (0.27–0.53)	<0.0001
Adjusted HR (95% CI)	Reference	0.37 (0.26–0.52)	<0.0001
ECC			
Observed person-years	47,825.8	50,286.9	
Incident cases	52	24	
Incidence rate †	1.09 (0.83–1.43)	0.48 (0.32–0.71)	
Crude HR (95% CI)	Reference	0.45 (0.28–0.73)	0.0011
Adjusted HR (95% CI)	Reference	0.44 (0.27–0.72)	0.0010
Pancreatic cancer			
Observed person-years	47,785.9	50,273.8	
Incident cases	101	61	
Incidence rate †	2.11 (1.74–2.57)	1.21 (0.94–1.56)	
Crude HR (95% CI)	Reference	0.58 (0.43–0.80)	0.0009
Adjusted HR (95% CI)	Reference	0.58 (0.42–0.79)	0.0007
Ampulla Vater cancer			
Observed person-years	47,829.0	50,289.0	
Incident cases	49	15	
Incidence rate †	1.02 (0.77–1.36)	0.3 (0.18–0.49)	
Crude HR (95% CI)	Reference	0.30 (0.17–0.53)	<0.0001
Adjusted HR (95% CI)	Reference	0.30 (0.17–0.53)	<0.0001
Lung cancer			
Observed person-years	47,765.7	50,171.8	
Incident cases	135	96	
Incidence rate †	2.83 (2.39–3.35)	1.91 (1.57–2.34)	
Crude HR (95% CI)	Reference	0.88 (0.63–1.24)	0.4753
Adjusted HR (95% CI)	Reference	0.86 (0.61–1.21)	0.3737

† per 100 person-years. ICC, Intrahepatic cholangiocarcinoma. ECC, Extra-hepatic cholangiocarcinoma. aHR, adjusted hazard ratio, the covariates included index year, sex, age, urbanization, unit type of insured, and co-morbidities. Competing aHR, competing adjusted hazard ratio was estimated by the Fine and Gray sub-distribution hazard function.

**Table 3:** Sensitivity analysis by moving the time 0 as +24 from index date.

	Cholecystectomy aHR (95% CI)
	Excluded Patients Followed <24 months
Incidence rate	
Cholangiocarcinoma	0.30 (0.17–0.51)
ICC	0.44 (0.25–0.75)
ECC	0.46 (0.18–1.14)
Pancreatic cancer	0.84 (0.47–1.48)
Ampulla Vater cancer	0.48 (0.16–1.40)
Lung cancer	1.05 (0.65–1.68)

ICC, Intrahepatic cholangiocarcinoma. ECC, Extra-hepatic cholangiocarcinoma. aHR, adjusted hazard ratio. The covariates included index year, sex, age, urbanization, unit type of insured, and co-morbidities.

## Data Availability

Datasets from the National Health Insurance Research Database are available through a request to the Health and Welfare Data Science Center (HWDSC). However, the data are not publicly available due to the privacy of research participants. We are unable to share the data sets and code lists on request. For this study, the application number is H111231, which was registered in HWDSC.

## Supplementary Materials

The following supporting information can be downloaded at https://www.mdpi.com/article/10.3390/cancers16050977/s1. Table S1: Baseline characteristics among original study groups (before propensity score matching); Table S2: definition of co-morbidities and study outcome; Table S3: Risks of cancer incidence among patients with EST/EPBD before propensity score matching; Table S4: Sensitivity analysis by moving the time 0 as +24 from index date before propensity score matching.
