# Supplementary material for: Association between Cholecystectomy and the Incidence of Pancreaticobiliary Cancer after Endoscopic Choledocholithiasis Management
Source: Cancers (Basel). 2024 Feb 28;16(5):977. doi: 10.3390/cancers16050977 (PMC10930920; doi:10.3390/cancers16050977)
Supplement: Supplementary file 1 [file cancers-16-00977-s001.zip › cancers-2841302-supplementary.pdf]

Supplementary Table S1. Baseline characteristics among original study groups (before propensity score matching)

	Non-Cholecystectomy	Cholecystectomy	ASD
n	33068	13413	
Index year			0.031
2011,2012	7592 (22.96%)	2915 (21.73%)	
2013,2014	8831 (26.71%)	3590 (26.77%)	
2015,2016	10368 (31.35%)	4207 (31.37%)	
2017,2018	6277 (18.98%)	2701 (20.14%)	
Sex			0.029
Male	17856 (54.00%)	7071 (52.72%)	
Female	15212 (46.00%)	6342 (47.28%)	
Age			0.423
<50	5193 (15.70%)	3714 (27.69%)	
50-60	4753 (14.37%)	2512 (18.73%)	
60-70	6499 (19.65%)	2988 (22.28%)	
≥70	16623 (50.27%)	4199 (31.31%)	
**Urbanization**			0.150
High urbanization	8604 (26.02%)	3896 (29.05%)	
Moderate urbanization	9751 (29.49%)	4207 (31.37%)	
Developing town	5329 (16.12%)	2097 (15.63%)	
General town	5149 (15.57%)	1853 (13.81%)	
Aged town	1107 (3.35%)	368 (2.74%)	
Agriculture town	1876 (5.67%)	639 (4.76%)	
Village	1252 (3.79%)	353 (2.63%)	
**Insured category**			0.219
Government	1755 (5.31%)	730 (5.44%)	
Privately held company	14494 (43.83%)	7189 (53.60%)	
Agricultural organizations	8125 (24.57%)	2391 (17.83%)	
Low-income	369 (1.12%)	103 (0.77%)	
Non-labor force	7876 (23.82%)	2778 (20.71%)	
Others	449 (1.36%)	222 (1.66%)	
**Co-morbidity**			
Chronic hepatitis B	2739 (8.28%)	1147 (8.55%)	0.010
Chronic hepatitis C	1673 (5.06%)	467 (3.48%)	0.078
Helicobacter infection	933 (2.82%)	494 (3.68%)	0.049
Diabetes mellitus	12556 (37.97%)	4222 (31.48%)	0.137
CKD	5547 (16.77%)	1273 (9.49%)	0.217
Congenital cystic disease of liver	343 (1.04%)	91 (0.68%)	0.039
Inflammatory bowel diseases	1072 (3.24%)	436 (3.25%)	0.001
Liver cirrhosis	2283 (6.90%)	513 (3.82%)	0.137

Supplementary Table S2. Definition of co-morbidities and study outcome.

Variable	Definition by ICD-9 codes
**Co-morbidity**	Records in NHIRD
Chronic hepatitis B	V02.61, 070.20, 070.22, 070.30 and 070.32
Chronic hepatitis C	V02.62, 070.41, 070.44, 070.51 and 070.54
Helicobacter infection	041.86
Diabetes mellitus	250.x
CKD	585.x
Congenital cystic disease of liver	751.62, 751.69
Clonorchis and opisthorchis	121.0, 121.1
Inflammatory bowel diseases	555.x, 556.x
Liver cirrhosis	571.2 571.5 571.6
**Outcome**	Records in cancer registry database
Intrahepatic cholangiocarcinoma (ICC)	155.1
Extra-hepatic cholangiocarcinoma (ECC)	156.1
Cholangiocarcinoma, not definite site	156.9
Pancreatic cancer	157.x
Ampulla Vater cancer	156.2

Supplementary Table S3. Risks of cancer incidence among patients with EST / EPBD before propensity score matching

	Non-Cholecystectomy	Cholecystectomy	p value
**Cholangiocarcinoma**			
Observed person-years	114025.9	50481.2	
Incident cases	464	61	
Incidence rate†	4.07(3.72-4.46)	1.21(0.94-1.55)	
Crude HR (95% CI)	Reference	0.31(0.23-0.40)	<.0001
Adjusted HR (95% CI)	Reference	0.36(0.28-0.47)	<.0001
Competing aHR (95% CI)	Reference	0.38(0.29-0.49)	<.0001
**ICC**			
Observed person-years	114146.3	50495.7	
Incident cases	326	47	
Incidence rate†	2.86(2.56-3.18)	0.93(0.70-1.24)	
Crude HR (95% CI)	Reference	0.33(0.25-0.45)	<.0001
Adjusted HR (95% CI)	Reference	0.40(0.29-0.54)	<.0001
Competing aHR (95% CI)	Reference	0.42(0.31-0.57)	<.0001
**ECC**			
Observed person-years	114307.1	50506.3	
Incident cases	147	24	
Incidence rate†	1.29(1.09-1.51)	0.48(0.32-0.71)	
Crude HR (95% CI)	Reference	0.38(0.25-0.59)	<.0001
Adjusted HR (95% CI)	Reference	0.46(0.30-0.71)	0.0005
Competing aHR (95% CI)	Reference	0.48(0.31-0.75)	0.0012
**Pancreatic cancer**			
Observed person-years	114203.9	50493.3	
Incident cases	275	61	
Incidence rate†	2.41(2.14-2.71)	1.21(0.94-1.55)	
Crude HR (95% CI)	Reference	0.52(0.39-0.68)	<.0001
Adjusted HR (95% CI)	Reference	0.56(0.43-0.75)	<.0001
Competing aHR (95% CI)	Reference	0.59(0.44-0.78)	0.0003
**Ampulla Vater cancer**			
Observed person-years	114304.8	50508.4	
Incident cases	131	15	
Incidence rate†	1.15(0.97-1.36)	0.30(0.18-0.49)	
Crude HR (95% CI)	Reference	0.27(0.16-0.46)	<.0001
Adjusted HR (95% CI)	Reference	0.30(0.18-0.51)	<.0001
Competing aHR (95% CI)	Reference	0.31(0.18-0.53)	<.0001
**Lung cancer**			
Observed person-years	114036.3	50392.3	
Incident cases	378	96	
Incidence rate†	3.31(3-3.67)	1.91(1.56-2.33)	
Crude HR (95% CI)	Reference	0.71(0.54-0.95)	0.0200

Adjusted HR (95% CI)	Reference	0.91(0.69-1.21)	0.5282
Competing aHR (95% CI)	Reference	0.99(0.74-1.32)	0.9428

† per 1000 person-years

ICC, Intrahepatic cholangiocarcinoma

ECC, Extra-hepatic cholangiocarcinoma

aHR, adjusted hazard ratio, the covariates included index year, sex, age, urbanization, unit type of insured, and co-morbidities.

Competing aHR, competing adjusted hazard ratio was estimated by Fine and Gray subdistribution hazard function

Supplementary Table S4. Sensitivity analysis by moving the time 0 as +24 from index date before propensity score matching

	aHR (95% CI)
	**Excluded patients followed <24 months**
Incidence rate	
Cholangiocarcinoma	0.34(0.21-0.56)
ICC	0.48(0.29-0.78)
ECC	0.57(0.25-1.29)
Pancreatic cancer	0.70(0.50-1.28)
Ampulla Vater cancer	0.53(0.20-1.41)
Lung cancer	1.11(0.76-1.62)

ICC, Intrahepatic cholangiocarcinoma

ECC, Extra-hepatic cholangiocarcinoma

aHR, adjusted hazard ratio, the covariates included index year, sex, age, urbanization, unit type of insured, and co-morbidities.
